# Food-specific IgG4-guided diet elimination improves allergy symptoms in children

**DOI:** 10.3389/fimmu.2024.1281741

**Published:** 2024-02-14

**Authors:** Boyun Yang, Hanxiao Yu, Wo Yao, Ran Diao, Bohui Li, Yongfang Wang, Ting Li, Liuya Ge, Yingying Hu, Huiying Wang

**Affiliations:** ^1^ Department of Allergy, Second Affiliated Hospital of Zhejiang University School of Medicine, Hangzhou, Zhejiang, China; ^2^ Clinical Research Center, Second Affiliated Hospital of Zhejiang University School of Medicine, Hangzhou, Zhejiang, China; ^3^ Outpatient Care Department, Second Affiliated Hospital of Zhejiang University School of Medicine, Hangzhou, Zhejiang, China

**Keywords:** food-specific IgG4, children, allergy, diet elimination, IgE

## Abstract

Allergic diseases in children are major public health concerns due to their widespread and rising prevalence. Food-specific immunoglobulin G4(FS-IgG4) has been detected in patients with allergic diseases, but its clinical significance is still debated. In the present study, 407 children with allergic diseases were recruited and categorized into three groups according to the different systems involved: the respiratory system group, the skin system group, and a multiple system group, with the collection of clinical symptoms and serum antibodies, including total immunoglobulin E (IgE), house dust mite (HDM) IgE, food-specific IgE (FS-IgE), and FS-IgG4. Part of these patients were followed up with the intervention of FS-IgG4-guided diet elimination with or without add-on probiotics supplement. The analysis at baseline revealed distinct serum levels of different antibodies. The positive rate of FS-IgG4 in all groups was more than 80%, and the proportion of total IgE and FS-IgG4 both positive in the multi-system group was the highest (p=0.039). Egg and milk were the foods with the highest positive rate of FS-IgG4 in all groups. After diet elimination for more than 3 months, serum FS-IgG4 in children significantly decreased (P<0.05) along with the improvement of clinical symptoms, regardless of the add-on of probiotics. However, the intervention did not impact the serum levels of total IgE, FS-IgE, and HDM IgE. There was no further decrease of serum FS-IgG4 level in children followed up for more than 1 year, which may be related to noncompliance with diet elimination. Multivariate regression analysis revealed that the decline of serum FS-IgG4 was an independent predictable factor for the improvement of clinical symptoms (adjusted OR:1.412,95%CI 1.017–1.96, p=0.039). The add-on of probiotics showed less efficiency in reducing the FS-IgG4 level in more patients with relief of clinical symptoms. Our results confirmed the correlation between FS-IgG4 and allergic diseases, and the decreased FS-IgG4 could be a useful predictor for the improvement of allergic symptoms. FS-IgG4-guided diet elimination is an efficient treatment for allergic diseases. Our study adds solid data to the clinical significance of FS-IgG4 in allergic diseases.

## Introduction

Allergic diseases have become some of the most prevalent chronic conditions affecting children worldwide. The prevalence of allergic diseases in children has increased dramatically over the past thirty years, as evidenced by three-phase surveys conducted by the International Study of Asthma and Allergies in Childhood (ISAAC) (1, 2). Pediatric allergies in China have also become a rising burden alongside rapid economic development and urbanization (3). Consequently, the prevention and intervention of pediatric allergies present a significant challenge for clinical practitioners. Management of allergic diseases extends beyond medication, with considerations such as food management proving to be crucial.

In the typical atopic march, atopic dermatitis (AD) often serves as the initial manifestation of allergy in infancy, followed by staggered occurrences of food allergy, allergic rhinitis, and allergic asthma (4). Food allergy is defined as an adverse immune response to food proteins and represents a spectrum of clinicopathologic manifestations, including gastrointestinal disturbances, hives, eczema, and airway inflammation, ranging in severity from mild to life-threatening (5). It can be categorized into three types: immunoglobulin E (IgE) mediated, non-IgE mediated, or mixed (6). IgE-mediated food allergy is typically characterized by exposure to very small amounts of allergic foods triggering clinical symptoms within minutes to hours after ingestion (5, 7). In contrast, non-IgE mediated food allergy has a delayed onset of symptoms, often presenting chronically, making the association with the specific allergens obscure and challenging to diagnose (7). Manifestations primarily include skin reactions (such as AD, contact dermatitis, and herpetiformis), respiratory reactions (such as Heinner syndrome), or gastrointestinal reactions such as eosinophilic esophagitis (EOE) (8). Mixed food allergies involve both IgE-dependent and IgE-independent pathways.

Apparently, IgE-mediated food allergies are widely recognized and feared by those affected. It is generally accepted that food can induce various forms of allergic reactions, from urticaria to asthma and, in urgent conditions, anaphylaxis, through specific IgE-mediated mast cell degranulation. Therefore, food-specific IgE (FS-IgE) is conventionally used as a clinical screening test for food allergy or food-related reactions. In fact, mast cell degranulation can also be activated via immunoglobulin G (IgG). Apart from FcϵRI, mast cells and basophils in humans and mice also express Fcγ receptors (FcγRs) that bind to IgG antibodies. These IgG antibodies have been shown to activate mast cells even before the discovery of IgE (9). Compared to IgE, IgG antibodies are more complex in structure and biology, and they have four subclasses: IgG1, IgG2, IgG3, and IgG4 (10). Among these, food-specific IgG4 (FS-IgG4) has been considered a potential clinical indicator for allergic symptoms (11).

The detection of FS-IgG4 appeared in the 1970s (12) and was soon applied in clinical work. However, the significance of IgG4 in allergic diseases is still controversial. It was not recommended for the diagnosis of food allergy in the most influential guidelines on food allergy, including those from the European Academy of Allergy and Clinical Immunology (EAACI) (13), the National Institute of Allergy and Infectious Diseases (NIAID) (14) and the American Academy of Allergy and Immunology Position Statement (AAAI) (15). These opposing opinions have clearly hindered the development of its application in clinical work until the studies on EOE changed the perception. Subsequent evidence has shown that FS-IgG4 is closely correlated with allergic diseases, including allergic rhinitis, asthma, atopic dermatitis, and chronic rhinosinusitis (16–19).

In the current study, we conducted a retrospective cross-sectional analysis utilizing data obtained from allergic patients aged 0-14 years. We assessed the positivity rates of total IgE, house dust mite (HDM) IgE, FS-IgE, and FS-IgG4 in various allergic systemic diseases. Furthermore, we identified and selected patients who underwent treatment involving FS-IgG4-guided diet elimination, with or without probiotics, for a duration exceeding 3 months and compared changes in clinical symptoms before and after treatment. Our investigation delves into pivotal questions, probing the relationships between FS-IgG4 and allergic diseases, and shedding light on the role of FS-IgG4 in allergic symptoms. The implementation of diet elimination guided by FS-IgG4 holds significant clinical relevance for controlling allergic symptoms in children. This study not only resolves existing uncertainties regarding FS-IgG4 but also provides novel clinical evidence for future research on its immunological mechanisms.

## Methods

### Study design and patients’ selection

**Figure 1:**
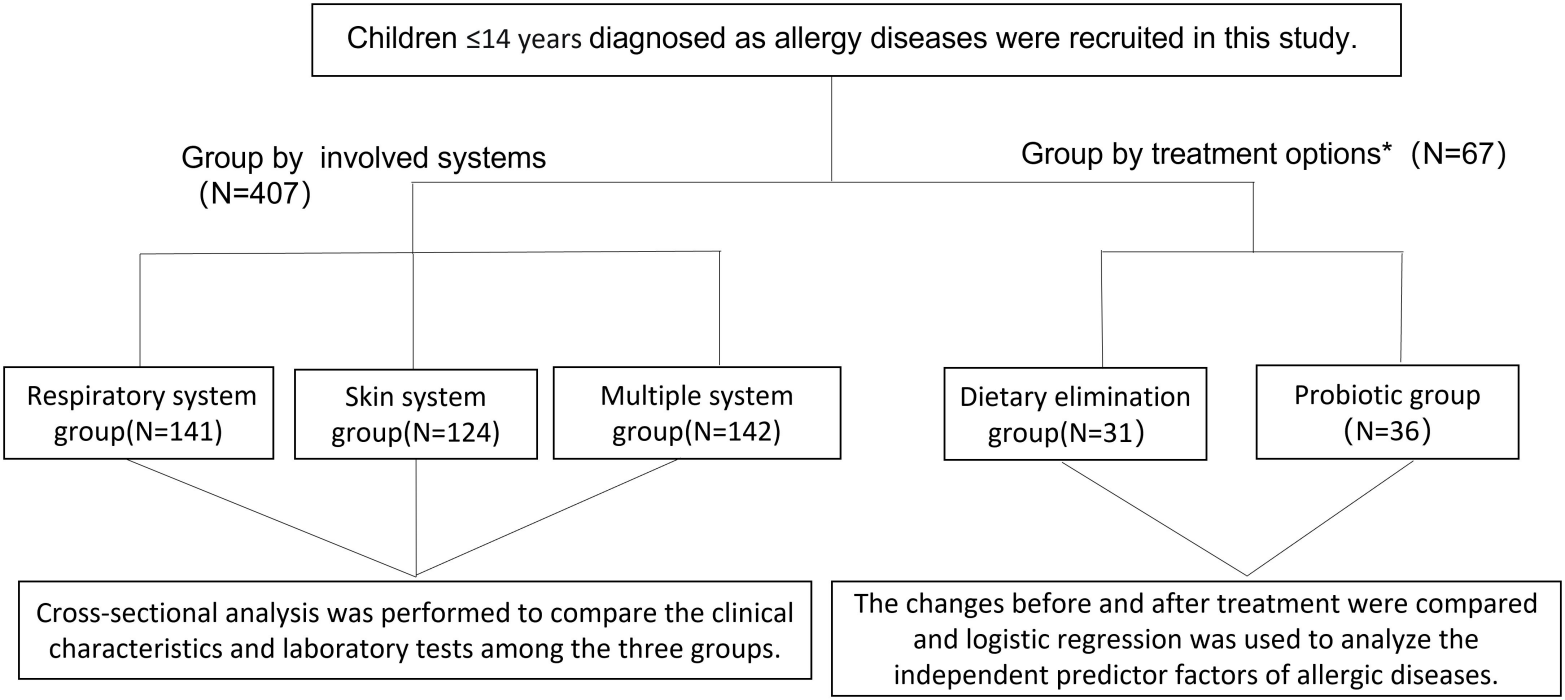
Flow chart of study participants. *Patients who were FS-IgG4 positive at initial diagnosis and had follow-up records for more than 3 months of treatment were selected.

This retrospective observational study aimed to evaluate the clinical significance of serum total IgE, HDM IgE, FS-IgE, and FS-IgG4 antibodies in the treatment of allergic diseases in children. Electronic medical records (EMR) of all patients with allergy diseases treated at the Department of Allergy, the Second Affiliated Hospital of Zhejiang University School of Medicine from January 2018 to December 2020 were collected and evaluated. The inclusion criteria were children aged 0-14 years who fulfilled the ARIA guideline for allergic rhinitis and/or GINA guideline for asthma (20, 21), the International Consensus (ICON) guideline for conjunctivitis (22), the EAACI/GA2 LEN/EDF/WAO guideline for urticaria (23), the diagnostic criteria by Hannifin and Rajka for atopic dermatitis (24), and with the data of serum total IgE, HDM IgE, FS-IgE, and FS-IgG4 tested simultaneously. Patients with autoimmune diseases were excluded. Ultimately, 407 patients were recruited for the analysis. Among them, 67 patients underwent dietary elimination guided by FS-IgG4 and supplemented with/without probiotics. After more than 3 months of treatment, serum total IgE, HDM IgE, FS-IgE, and FS-IgG4 were retested, and clinical manifestations were reevaluated. The study flowchart is shown in **Figure 1**.

### Study groups

According to the system affected, patients were categorized into three groups: the group with involvement of the respiratory system (including rhinitis, asthma, and conjunctivitis), the group with involvement of the skin system (including urticaria and atopic dermatitis), and the group with multiple systems involvement (those with a combination of symptoms from different systems). Furthermore, patients with a follow-up history were divided into two groups based on their treatment regimen: those who underwent FS-IgG4-guided diet elimination for more than 3 months were assigned to the dietary elimination group, and those who underwent diet elimination combined with probiotic were assigned to the probiotic group.

### Study variables and laboratory testing

Clinical characteristics and demographic profiles were obtained from the hospital’s EMR system. Peripheral blood samples were taken from patients at the time of the initial presentation and during treatment according to the patients’ condition. All laboratory tests were carried out in our hospital laboratory. Serum total IgE was measured by colloidal gold immunochromatography (Siemens Healthcare Diagnostics Products Limited, United Kingdom), HDM IgE and fifteen species of FS-IgE, including egg, milk, beef, crab, shrimp, cashews, mangoes, lamb, shellfish, lobster/scallops, cod, salmon, peanuts, beans, and pineapple, were detected by immunoblotting (Hangzhou Zheda Dixun Biological Gene Engineering Co., Ltd, China). All of these were expressed in international units per unit volume (IU/ml). Levels of total IgE value>100IU/ml, HDM IgE and FS-IgE value>0.35 IU/ml were considered positive. Ten species of FS-IgG4, including cod, egg, milk, beef, shrimp, soy, wheat, chicken, crab, and mushroom, were determined by enzyme-linked immunosorbent assay (Hangzhou Zheda Dixun Biological Gene Engineering Co., Ltd, China). The concentration of FS-IgG4 was divided into 4 grades: negative (−, <250 U/ml), weakly positive (+,250-500U/ml), positive (++,500-1000U/ml), strong positive (+++,>1000U/ml).

### Diet elimination and probiotic supplement

Sixty-seven patients underwent diet elimination based on the result of FS-IgG4. Foods determined as ++ or +++ were completely forbidden to eat, and + foods were reduced in frequency of intake (eating the food at an interval of more than 4 days). Among them, 36 of the patients were supplemented with probiotics in addition to dietary elimination. The viable bacteria were composed of *Lactobacillus paracasei LP33, Lactobacillus fermentum GM090* and *Lactobacillus acidophilus GMNL-185*(GenMont Biotec Inc.). The above treatment time was required to last more than 3 months.

### Outcome measure

Demographic data, serum level of total IgE, HDM IgE, FS-IgE, and FS-IgG4 from the three groups involving different systems were analyzed initially and at follow-up visits after varying periods of intervention (3-6 months, 6-9 months, 9-12 months, or over 12 months) with diet elimination, with/without probiotic supplementation. Laboratory-related indicators, such as total IgE and HDM IgE, were numerically compared. Due to the low positive frequency of FS-IgE, only the overall positive rate was used for statistical comparison. For FS-IgG4, the test data may exceed the kit reference range. The intervention of diet elimination or probiotic supplement caused distinct changes in different FS-IgG4; for example, one may decrease, but another may increase. Therefore, we designed a point system for statistical testing. We used 1 point to stand for one kind of elevated FS-IgG4, so patients’ FS-IgG4 status was scored according to the number of foods with positive FS-IgG4. The total was then used to compare the difference between pre- and post-intervention. The same scoring method was applied to FS-IgE changes before and after treatment. Furthermore, the improvement of clinical symptoms was classified based on the subjective feelings of patients before and after treatment by comparing the numbers in the dietary elimination and probiotic group showing significant improvement (‘better ‘or ‘excellent’), and those with no significant change (‘slightly worse ‘, ‘no change’, or ‘slightly better’). Finally, we explored independent factors influencing the clinical manifestations of allergic diseases through regression analysis.

### Statistical analysis

For all patients, continuous data following a normal distribution were expressed as mean ± standard deviation and compared using single-factor analysis of variance (ANOVA). Non-normally distributed data were expressed as median (interquartile range) and compared using the Kruskal-Wallis test. Categorical variables were expressed as frequencies (percentages) and compared using the Chi-square or Fisher’s exact test. For patients before and after treatment, the paired t-test was used for normally distributed data, and the Wilcoxon signed-rank test was used for non-normally distributed data. The Student’s t-test was employed to compare normally distributed data, while the Mann-Whitney U test was used for non-normally distributed data between the two treatment groups. Logistic regression analysis was conducted to determine whether the changes in total IgE, HDM IgE, FS-IgE, and FS-IgG4 before and after treatment were independent predictors for improvement of allergic diseases in children. Variables with an adjusted p value<0.1 in the univariate analysis were subsequently evaluated using a multivariate logistic regression model. A p-value <0.05 was the criterion for statistical significance in this analysis. Statistical analysis of all data was performed using SPSS 26.0 (IBM Corp.) and GraphPad Prism 8.0.1.

## Results

### Demographic characteristics

A total of 407 patients, with an average age of 7.2 ± 3.6 years (range 8 months to 14 years), were recruited and divided into three groups as following: 141 patients in the group with respiratory system involvement, 124 in the skin system group and 142 in the group with multiple systems involved. The baseline characteristics of the three groups are shown in **Table 1**. There were no significant differences in gender, age, family history of allergy, or birth history among the three groups (p> 0.05). Patients with multiple system allergies had the highest serum total IgE level (p < 0.05), and the lowest level of HDM IgE was observed in patients of the skin system (p < 0.01). The positive rate of FS-IgE was only about 30%, and there was no significant difference among the three groups. However, the positive rate of FS-IgG4 was more than 80 percent, and no significant difference among the three groups was found.

**Table 1:** Clinical and demographic characteristics of the study population.

Characteristics	Respiratory system group	Skin system group	Multiple system group	P
**Patients, n**	141	124	142	
**Age (years), Mean ± SD**	7.4 ± 3.5	6.7 ± 3.9	7.3 ± 3.3	0.093
Gender, n (%)				
**Male**	97 (68.8)	97 (68.8)	83 (58.5)	0.065
**Female**	44 (31.2)	55 (44.4)	55 (44.4)	
Mode of birth, n (%)				
**Maternity leave**	74 (52.5)	77 (62.1)	78 (54.9)	0.194
**Cesarean**	60 (42.6)	45 (36.3)	56 (39.4)	
**Premature delivery**	7 (5.0)	2 (1.6)	8 (5.6)	
Family history of allergy, n (%)				
**Yes**	54 (38.3)	48 (38.7)	59 (41.5)	0.833
**NO**	87 (61.7)	76 (61.3)	83 (58.5)	
**Total IgE (IU/ml), Median (Q1-Q3)**	185.0 (68.13-480.5)	126.0 (49.7-328.0) ^#^	204.0 (78.8-549.0) ^#^	0.033*
**HDM IgE (IU/ml), Median (Q1-Q3)**	2.75 (0.34-9.05)	0.34 (0.25-1.98)	2.2 (0.34-9.7)	0.003**
**Positive rate of FS-IgE, n (%)**	52 (36.9)	30 (24.2)	41 (28.9)	0.073
**Positive rate of FS-IgG4, n (%)**	121 (85.8)	100 (80.6)	119 (83.8)	0.631

Values are presented as mean ± SD or as absolute numbers (percentage). Non-normally distributed data are expressed as median (interquartile range). (*p<0.05). Pairwise comparisons (respiratory system group vs. skin system group; respiratory system group vs. multiple system group or skin system group vs. multiple system group, ^#^p<0.05).

### The difference in total/FS-IgE and FS-IgG4 positive rates among the three groups

In the three groups, we measured the proportion of patients who were positive for both total/FS-IgE and FS-IgG4, positive for total/FS-IgE but negative for FS-IgG4, negative for total/FS-IgE but positive for FS-IgG4, and negative for both. From the distribution of each group, the proportion of patients with both total IgE and FS-IgG4 positive was the highest, and the proportion of both negative was the lowest. It is noteworthy that the proportion of patients with FS-IgG4 positive and total IgE negative was higher than that of patients with total IgE positive and FS-IgG4 negative. Meanwhile, we found that the distribution of proportions among the three groups was significantly different (p<0.05) (**Table 2**). Upon pairwise comparison between groups (according to the multiplicity test criteria), the proportion of patients with both positive results in the multiple system group was significantly higher than that in the skin system group (p=0.012). Different from total IgE, the positive rates of FS-IgE and FS-IgG4 were not significantly different among all groups (**Table 3**).

**Table 2:** The positive rates of serum total IgE and FS-IgG4 in children with allergic diseases involving different systems.

Groups	Total IgE (+) FS-IgG4 (+) (%, n)	Total IgE (+) FS-IgG4 (–) (%, n)	Total IgE (-) FS-IgG4 (+) (%, n)	Total IgE (-) FS-IgG4 (-) (%, n)	X^2^	P
**Respiratory system group**	58.2 (n=82)	9.9 (n=14)	27.7 (n=39)	4.3 (n=6)	13.36	0.039*
**Skin system group^#^**	51.6 (n=64)	9.7 (n=12)	29.8 (n=37)	8.9 (n=11)		
**Multiple system group^#^**	71.1 (n=101)	7.0 (n=10)	16.9 (n=24)	4.9 (n=7)		

Pairwise comparisons (respiratory system group vs. skin system group; respiratory system group vs. multiple system group or skin system group vs. multiple system group, ^#^ p<0.05). (*p<0.05).

**Table 3:** The positive rates of serum FS-IgE and FS-IgG4 in children with allergic diseases involving different systems.

Groups	FS-IgE (+) FS-IgG4 (+) (%, n)	FS-IgE (+) FS-IgG4 (-) (%, n)	FS-IgE (-) FS-IgG4 (+) (%, n)	FS-IgE (-) FS-IgG4 (-) (%, n)	X^2^	P
**Respiratory system group**	34.0 (n=47)	2.8 (n=5)	51.8 (n=73)	11.3 (n=16)	6.922	0.32
**Skin system group**	19.4 (n=24)	4.8 (n=6)	61.3 (n=76)	14.5 (n=18)		
**Multiple system group**	26.8 (n=37)	2.1 (n=5)	57.0 (n=80)	14.1 (n=20)		

### Comparison of FS-IgG4 positive rates among the three groups

The positive rate of each assayed FS-IgG4 of 10 kinds was analyzed and compared among the three groups. Detailed data are shown in **Table 4**. In all the groups, eggs had the highest positive rate, followed by milk, while mushrooms were not detected as positive. Meanwhile, we found that children with respiratory allergic diseases had the highest positive rate of milk, which was significantly different from that of the skin system (p<0.01). However, the positive rates of wheat and soybean in the multiple system group were the highest, which were significantly higher than that in the skin system group (p<0.05) and the respiratory system group (p<0.05) respectively.

**Table 4:** The positive rates of the FS-IgG4 in three groups. (*p<0.05).

Groups	Respiratory system group	Skin system group	Multiple system group	P
**Egg (%, n)**	81.6 (n=115)	75.8 (n=94)	79.6 (n=113)	0.51
**Milk (%, n)**	70.9 (n=100) ^#^	54.8 (n=100) ^#^	64.1 (n=91)	0.025*
**Wheat (%, n)**	19.1 (n=27)	12.1 (n=15) ^#^	26.1 (n=37) ^#^	0.0163*
**Gadus (%, n)**	18.4 (n=26)	14.5 (n=18)	25.4 (n=36)	0.0776
**Soybean (%, n)**	7.8 (n=11) ^#^	13.7 (n=17)	19.0 (n=27) ^#^	0.0224*
**Shrimp (%, n)**	3.2 (n=4)	3.2 (n=4)	6.3 (n=9)	0.2775
**Crab (%, n)**	2.1 (n=3)	3.2 (n=4)	6.3 (n=9)	0.2086
**Beef (%, n)**	2.8 (n=4)	2.4 (n=3)	4.9 (n=7)	0.4751
**Chicken (%, n)**	3.5 (n=5)	2.4 (n=3)	6.3 (n=9)	0.2532
**Mushroom (%, n)**	0 (n=0)	0 (n=0)	0 (n=0)	–

Pairwise comparisons (respiratory system group vs. skin system group; respiratory system group vs. multiple system group or skin system group vs. multiple system group, ^#^ p<0.05).

### Outcome before and after treatments

**Figure 2:**
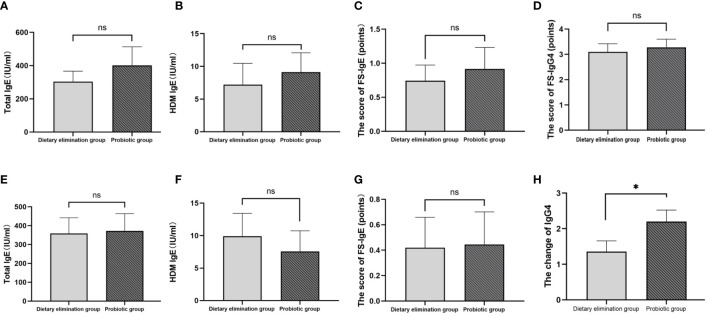
Comparison of total IgE, HDM IgE, FS-IgE, and FS-IgG4 between the dietary elimination group and the probiotic group before **(A-D)** and after treatment **(E-H)** (*p<0.05). ns, no significant.

**Figure 3:**
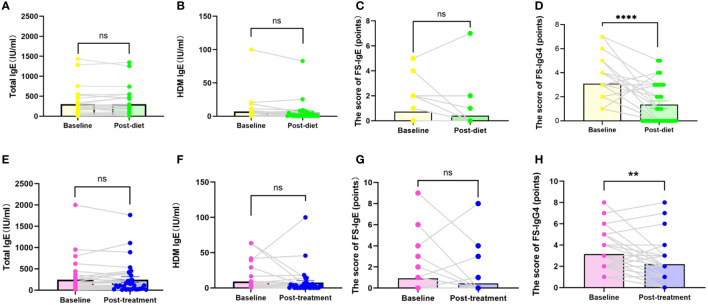
Comparison of total IgE, FS-IgE, HDM IgE, and FS-IgG4 in baseline and after treatment in the dietary elimination group **(A-D)** and the probiotic group **(E-H)**. **P<0.01; ****P<0.0001; ns, no significant.

**Figure 4:**
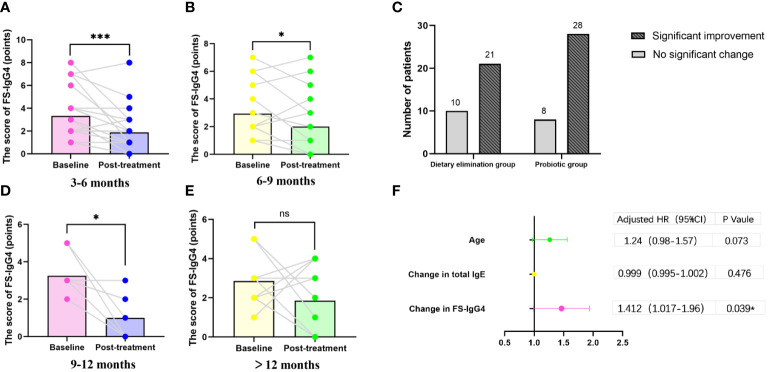
For all the treated patients (N=67), FS-IgG4 changes according to different follow-up intervals **(A, B, D, E)** (*p<0.05). The proportion of patients with improvement in clinical symptoms in both groups before and after treatment **(C)** (*p<0.05); Forest plot of outcomes in study population (N=67). Outcomes are presented as odds ratios with 95% confidence intervals. OR<1 favors no significant remission, OR>1 favors alleviating. Univariate analysis demonstrated children with allergies whose FS-IgG4 decreased more had a higher rate of clinical remission **(F)**. (*p<0.05); ***P<0.001; ns, no significant.

Of the 407 participants, 67 underwent at least one follow-up evaluation and repeated laboratory testing under diet elimination guided by FS-IgG4 with or without probiotic supplements. The follow-up interval of patients in both groups before and after treatment was more than 3 months, with the longest follow-up interval being two and a half years. There were no significant differences in total IgE, HDM IgE, FS-IgE, and FS-IgG4 between the dietary elimination group and the probiotic group before treatment. However, after treatment, there was a significant difference in FS-IgG4 between the two groups, and the decrease was more pronounced in the dietary elimination group. Total IgE, FS-IgE, and HDM IgE remained unchanged (**Figure 2**). In addition, no significant differences before and after treatment were noted with regard to the changes in the total IgE, FS-IgE, and HDM IgE between the dietary elimination group and probiotic group. Yet, we found a significant decrease in FS-IgG4 before and after treatment in both groups. Comparatively, a more significant decrease was observed in the dietary elimination group (**Figure 3**). Moreover, the patients were assigned to four groups according to the follow-up interval, which was 3-6 months, 6-9 months, 9-12 months, and > 12 months. At different follow-up intervals, FS-IgG4 decreased most significantly in patients who were followed up for 9-12 months, followed by 3-6 months and 6-9 months, while FS-IgG4 did not decrease significantly in patients longer than 12 months (**Figures 4A, B, D, E**). Similarly, the clinical symptoms of patients in both groups were improved dramatically. Although there were no significant differences between the two groups, the percentage of patients with a significant improvement was 67.7% (n=21) in the dietary elimination group and 77.8% (n=28) in the probiotic group, with the remainder having no significant responses (**Figure 4C**).

### The results of binary logistic regression analysis

In order to find the predictable index of the improvement of clinical symptoms, we used a multivariate logistic regression model to test different variables including age, gender, birth history, family history, use of probiotics supplement or not, and the changes of total IgE, HDM IgE, FS-IgE, and FS-IgG4. The results showed that the change in FS-IgG4 was an independent predictor for significant improvement of clinical symptoms in children with allergies (adjusted OR: 1.412,95% CI 1.017–1.96, p=0.039; **Figure 4F**).

## Discussion

The current study is the first clinical investigation to confirm the clinical correlation of FS-IgG4 antibodies with allergic diseases through the intervention of FS-IgG4-guided diet elimination. We observed elevated serum FS-IgG4 levels in allergies of different systems and analyzed the expression of different FS-IgG4 cations for 10 common foods. Moreover, we further followed up on these patients for at least 3 months with the intervention and found that FS-IgG4-guided diet elimination significantly improved the allergic symptoms.FS-IgG4 also emerged as an independent predictor for clinical improvement.

In our study, we observed a significantly higher positive rate of FS-IgG4 compared to FS-IgE in allergic children. Measurement of total IgE, HDM IgE, FS-IgE, and FS-IgG4 revealed that most patients exhibited positive total IgE and FS-IgG4, with a very low positive rate of FS-IgE. This finding aligns with previous studies (14), in spite of the rapid increase in food allergy in children. Our results suggest a potentially more crucial role for FS-IgG4 in allergic rhinitis/asthma or urticaria and AD among Chinese children, indicating that the immediate response mediated by FS-IgE might not be the primary cause of these chronic allergies. Furthermore, the dual positivity of IgE and FS-IgG4 is significantly higher in the group of multiple systems, implying a correlation between the number of systems affected and the extent of immune reactions. Notably, the positive ratio of FS-IgG4(+) total IgE (–) is higher than FS-IgG4 (–) total IgE (+). Regardless of the system involved, more than 80% of allergic children exhibit a positive rate of FS-IgG4, while the positive rates of total IgE and FS-IgE are comparatively lower. Previous research has often explained the rapid development of FS-IgG4 in early-stage allergic children exposed to food as a normal physiological response (25, 26)., leading to its clinical significance being overlooked. However, in healthy children, the increase in serum IgG4 is very gradual, reaching adult levels only around puberty (27). Therefore, any elevation beyond normal adult levels can be considered potentially impactful.

Further analysis of the positive rates of FS-IgG4 for different foods revealed that FS-IgG4 levels were highest for egg and milk. This is likely attributed to greater exposure to these two types of foods during childhood. Milk is also the leading cause of food allergies (14). This meets the explanation of a previous study by *Millers etc.* Their research on EOE demonstrated a close association with IgG4 antibody levels in milk and wheat (28). Given that many children with allergic diseases continue to consume large amounts of eggs, milk, wheat products, and soy milk for growth needs, it is not surprising that the corresponding rise in FS-IgG4 antibodies was significant. Researches confirm that continuous exposure to allergens induces eosinophilic inflammation in the esophagus with locally significant IgG4 deposition in patients with EOE (29) and patients receiving oral immunotolerance therapy (30). Nevertheless, dietary elimination as a treatment for EOE has been shown to have positive effects in both adults and children (31).

The intervention of diet elimination guided by FS-IgG4 in our study also demonstrated a significant improvement in symptoms correlation with serum indices, in accordance with previous research on EOE. Serum FS-IgG4 decreased significantly after diet elimination, accompanied by a substantial improvement in symptoms. Regression analysis indicated that the reduction in FS-IgG4 was an independent predictor affecting the degree of clinical symptom remission in allergic children. Neither total IgE nor FS-IgE changed in spite of the dramatic improvement in symptoms, further confirming that FS-IgG4 played a more critical role in these allergic reactions, whether in the skin or respiratory systems. These observations were in keeping with very recent studies that eliminated allergic foods based on FS-IgG antibodies in patients with irritable bowel syndrome, Crohn’s disease, and migraine, all of which demonstrated relief of related symptoms (32–36).

Compared to another intervention of probiotic supplements as add-on strategies, the changes of FS-IgG4 are more pronounced in children with diet elimination. However, the relief ratio of clinical symptoms of allergies is more efficient in the probiotic group, with 77.8% compared to 67.7% in the diet elimination group. This may result from the multiple impacts of probiotics in relieving clinical symptoms besides the modulation of antibody production. The relief of clinical symptoms of probiotics led to less strict diet elimination, resulting in a smaller decrease in FS-IgG4 compared to children undergoing only diet elimination. The changes in serum FS-IgG4 within a year of follow-up duration provide interesting evidence to verify this hypothesis. We observed a lasting decrease in FS-IgG4 with the duration of diet elimination, followed by an increase at the 1-year time point. We speculate that children who experience sufficient relief under short-term diet elimination often discontinue therapy and reintroduce the food due to concerns about nutritional deficiencies.

In this study, we have observed a close relationship between FS-IgG4 and allergic symptoms in children. After dietary elimination guided by FS-IgG4, clinical symptoms improved significantly, indicating that the occurrence of allergic symptoms in children is induced by both IgG and IgE and may even occur independently of IgE. In classical allergic sensitization, IgE-producing plasma cells are generated, and initial symptoms may stem from IgG-producing B cells reacting to allergens. The IgG-to-IgE class switching process primes mast cells. However, numerous allergic reactions can occur independently of allergen-specific IgE, even in the absence of total IgE. IgG, contributing to Th2 polarization, enhances allergic responses. Allergy processes go beyond specific IgE, with IgG influencing atopy, clinical symptoms, and the resolution of allergies. Importantly, the pattern of IgG-producing plasma cells in atopic children signifies crucial events leading to enduring mast cell sensitization in allergies (37). In previous studies, it has been detected that IgE and IgG antibodies share similar portions, indicating common antigen secretion triggers that can bind to similar antigenic epitopes, particularly in patients with peanut or milk allergies (38, 39). Similar results have also been validated in patients with wheat and soy allergies (40, 41). In the comorbidity analysis of epitope-specific antibodies in children with egg allergies, it was observed that nearly all elevated levels of epitope-specific IgG4 antibodies were associated with rhinitis. In a model adjusted for asthma and age, one IgG4 epitope exhibited a significant correlation (42). This evidence suggests the presence of IgE conversion mechanisms, including the production of IgG, in patients with food allergies. In both mice and humans, evidence suggests that allergies or inflammatory responses can be mediated by IgG (43, 44), indicating that the onset of allergies may be independent of IgE and instead triggered by IgG or alternative pathways.

In conclusion, serum FS-IgG4, but not FS-IgE, is found to be correlated with allergic diseases more significantly than previously recognized. Eggs and milk emerge as the most common allergens influencing the development of allergic symptoms. Diet elimination guided by FS-IgG4 proves to be an effective method for managing allergic diseases in children. Our study thus contributes solid data to the understanding of the role of FS-IgG4 in allergic diseases. Our findings hold the potential to advance the comprehension of the clinical significance of FS-IgG4 in allergic diseases and provide valuable insights for the diagnosis and treatment of pediatric allergic conditions.

## Data availability statement

The original contributions presented in the study are included in the article/supplementary material. Further inquiries can be directed to the corresponding author.

## Ethics statement

The studies involving humans were approved by the Ethics Committee of the second affiliated hospital of Zhejiang university school of medicine (No. 2022-0380). The studies were conducted in accordance with the local legislation and institutional requirements. Written informed consent for participation in this study was provided by the participants' legal guardians/next of kin. Written informed consent was obtained from the minor(s)' legal guardian/next of kin for the publication of any potentially identifiable images or data included in this article.

## Author contributions

BY: Conceptualization, Data curation, Formal analysis, Funding acquisition, Investigation, Methodology, Project administration, Software, Supervision, Validation, Visualization, Writing – original draft, Writing – review & editing. HY: Data curation, Formal analysis, Investigation, Methodology, Software, Writing – original draft, Writing – review & editing. WY: Conceptualization, Investigation, Methodology, Project administration, Writing – review & editing. RD: Investigation, Methodology, Project administration, Writing – review & editing. BL: Formal analysis, Investigation, Methodology, Writing – review & editing. YW: Investigation, Methodology, Writing – review & editing. TL: Data curation, Investigation, Writing – review & editing. LG: Data curation, Investigation, Writing – review & editing. YH: Data curation, Investigation, Writing – review & editing. HW: Conceptualization, Formal analysis, Funding acquisition, Investigation, Methodology, Supervision, Validation, Visualization, Writing – original draft, Writing – review & editing.
